# Temporary dense seismic network during the 2016 Central Italy seismic emergency for microzonation studies

**DOI:** 10.1038/s41597-019-0188-1

**Published:** 2019-09-25

**Authors:** Fabrizio Cara, Giovanna Cultrera, Gaetano Riccio, Sara Amoroso, Paola Bordoni, Augusto Bucci, Ezio D’Alema, Maria D’Amico, Luciana Cantore, Simona Carannante, Rocco Cogliano, Giuseppe Di Giulio, Deborah Di Naccio, Daniela Famiani, Chiara Felicetta, Antonio Fodarella, Gianlorenzo Franceschina, Giovanni Lanzano, Sara Lovati, Lucia Luzi, Claudia Mascandola, Marco Massa, Alessia Mercuri, Giuliano Milana, Francesca Pacor, Davide Piccarreda, Marta Pischiutta, Stefania Pucillo, Rodolfo Puglia, Maurizio Vassallo, Graziano Boniolo, Grazia Caielli, Adelmo Corsi, Roberto de Franco, Alberto Tento, Giovanni Bongiovanni, Salomon Hailemikael, Guido Martini, Antonella Paciello, Alessandro Peloso, Fabrizio Poggi, Vladimiro Verrubbi, Maria Rosaria Gallipoli, Tony Alfredo Stabile, Marco Mancini

**Affiliations:** 1 Istituto Nazionale di Geofisica e Vulcanologia, Sezione di Roma1, Roma, Italy; 2 Istituto Nazionale di Geofisica e Vulcanologia, Sezione di Roma1, Grottaminarda, Italy; 3 Università Degli Studi G. D’Annunzio di Chieti - Pescara, Pescara, Italy; 4 Istituto Nazionale di Geofisica e Vulcanologia, Sezione di Milano, Milano, Italy; 5 Istituto Nazionale di Geofisica e Vulcanologia, Sezione di Roma1, L’Aquila, Italy; 6 Consiglio Nazionale delle Ricerche - Istituto per la Dinamica dei Processi Ambientali, Milano, Italy; 7 Agenzia Nazionale per le nuove tecnologie, l’energia e lo sviluppo economico sostenibile, Roma, Italy; 8 Consiglio Nazionale delle Ricerche – Istituto di Metodologie per l’Analisi Ambientale, Tito Scalo, Italy; 9 Consiglio Nazionale delle Ricerche – Istituto di Geologia Ambientale e Geoingegneria, Monterotondo, Italy

**Keywords:** Seismology, Natural hazards

## Abstract

In August 2016, a magnitude 6.0 earthquake struck Central Italy, starting a devastating seismic sequence, aggravated by other two events of magnitude 5.9 and 6.5, respectively. After the first mainshock, four Italian institutions installed a dense temporary network of 50 seismic stations in an area of 260 km^2^. The network was registered in the *International Federation of Digital Seismograph Networks* with the code 3A and quoted with a *Digital Object Identifier* (10.13127/SD/ku7Xm12Yy9). Raw data were converted into the standard binary miniSEED format, and organized in a structured archive. Then, data quality and completeness were checked, and all the relevant information was used for creating the metadata volumes. Finally, the 99 Gb of continuous seismic data and metadata were uploaded into the INGV node of the *European Integrated Data Archive* repository. Their use was regulated by a *Memorandum of Understanding* between the institutions. After an embargo period, the data are now available for many different seismological studies.

## Abstract (summary)

**Table:** 

Measurement(s)	seismic activity • signal conversion function
Technology Type(s)	Sensor • Digitizer Device
Factor Type(s)	location
Sample Characteristic - Location	Italy

Machine-accessible metadata file describing the reported data: 10.6084/m9.figshare.9693602

## Background & Summary

Temporary seismic networks, installed few hours after high-magnitude earthquakes, are important tools for understanding the seismic phenomena under several perspectives^1,2^.

In particular, the continuous ground motion recordings, organized in a standard international format, can be used to:

- improve the ability to detect the seismicity as well as to follow its spatial-temporal evolution^3^;
- record small-to-large magnitude earthquakes by using different instrumentation (velocimeters and accelerometers). In particular, accelerometric recordings enrich the available accelerometric archives that are used for several purposes, such as improving the design methods and calibrating attenuation models for the ground motion parameters^4–7^;
- produce experimental data that can be used to estimate source properties^8,9^, crustal propagation and seismic site response^10–12^, also supporting civil protection activities^13^.

Among these aims, ground motion data recorded during seismic emergencies are used in microzonation studies to evaluate site response functions, aggravation factors, response spectra, and as benchmark against numerical simulations^14–16^.

In 2015 several Italian research institutions and university departments established the *Centro di microzonazione sismica e le sue applicazioni* (*Center for seismic microzonation and its applications*, hereinafter CentroMS; https://centromicrozonazionesismica.it/en), with the aims to provide scientific and technical support to the institutions involved in seismic microzonation and its applications, with particular reference to urban planning and management of seismic emergencies. During the 2016–2018 seismic sequence of Central Italy, CentroMS supported the largest and most complex study of post-event seismic microzonation in Italy through a coordinated activity of the Civil Protection Department on the behalf of the Italian Government, Regions and local administrations, involving professionals and the scientific community.

**Fig. 1:**
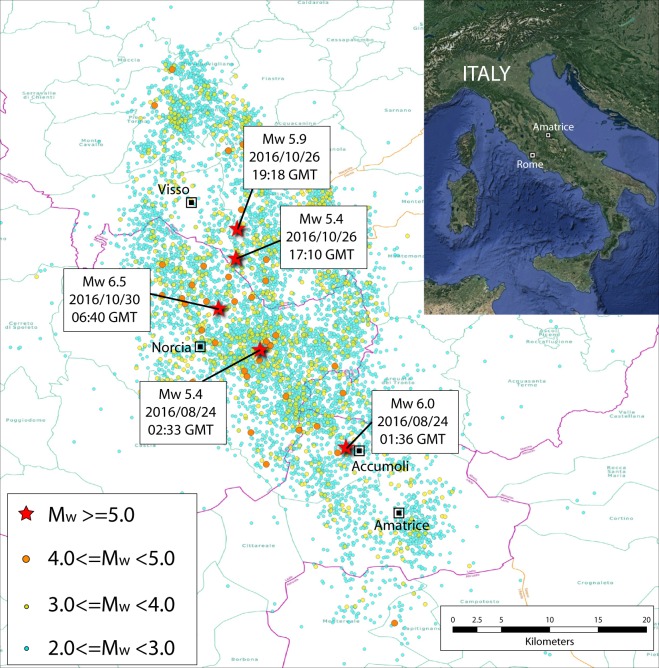
Central Italy seismic sequence recorded from August 24, 2016 to November 30, 2016. Epicentral position of all the events of moment magnitude (Mw) larger than 2 recorded during the cited time interval; five of them (red stars) had a Mw larger than 5.4. The inlet shows the map of Italy with the position of Amatrice. The epicentral localizations are from the INGV web service (http://terremoti.ingv.it/en/, last access June 2018).

The Central Italy sequence started on August 24, 2016, with a moment-magnitude (Mw) 6.0 earthquake localized close to the villages of Accumoli and Amatrice in the Apennines (Fig. 1), causing 299 victims and heavy damages. Accumoli and downtown Amatrice were completely destroyed^17^. A strong shock of Mw 5.9 occurred on October 26, while on the 30th of October, a Mw 6.5 earthquake was localized close to the village of Norcia, about 20 km NW of Amatrice; this latter earthquake is the most energetic event in the previous 35 years in Italy. As of May 2018, the sequence is still active and interests a 80 km long band all along the Apennines (Fig. 1).

**Fig. 2:**
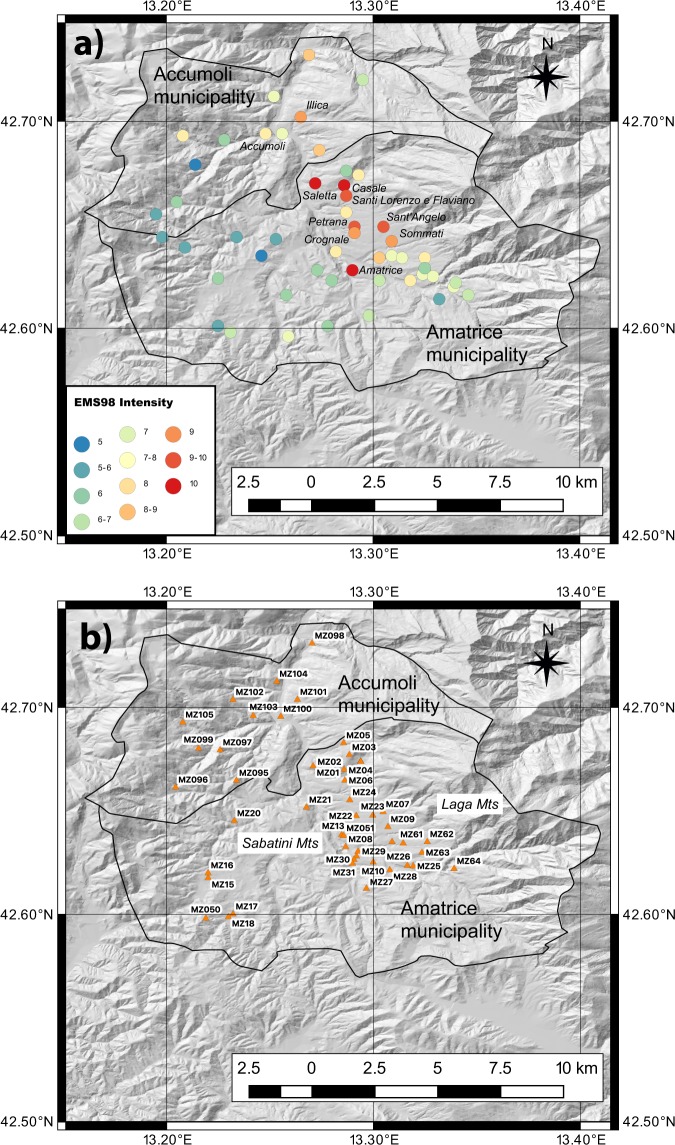
EMS-98 Intensity and stations location of network 3A installed at Accumoli and Amatrice municipalities. Digital elevation model of the Accumoli and Amatrice municipalities together with: (**a**) EMS-98 intensity for the hamlets in the two municipalities (the black lines indicate the limits) after the Mw 6.0 earthquake occurred on the 24th of August 2016. In the figure only the names of the villages reporting EMS-98 intensity larger than 9.0 are showed, as well as Accumoli which suffered intensity 8.0. (**b**) Temporary network 3A installed in the two municipalities.

After the first shock and with some updates due to the other strong earthquakes, CentroMS was in charge for carrying out a series of preparatory activities (e.g. geological and geophysical surveys, and earthquake monitoring) for seismic microzonation of the most damaged zones. Two of them were the municipalities of Amatrice and Accumoli (Fig. 2a, with the EMS-98 intensity^18^), which comprised tens of hamlets of different extension, number of inhabitants and level of damage. CentroMS then selected a subset of 65 hamlets to be investigated on the base of the level of damage and inhabitants.

In this paper, we present the temporary seismic network installed in the Amatrice and Accumoli municipalities (Fig. 2b) and the stages to prepare the large amount of data to be shared with the scientific community. The institutions involved in this study agreed to share the collected data in the Italian node of the European Integrated Data Archive (EIDA; http://eida.rm.ingv.it), defining a common data policy and quoting the temporary network with a digital object identifier (DOI).

The recorded data has been already analysed with standard techniques^19^ to produce synthetic reports (Supplementary Fig. 1) for the aims of the CentroMS, and therefore considerable information have been used for the seismic microzonation studies of the involved municipalities.

This large dataset is really valuable, because it is characterized by a very dense network deployed in the most damaged hamlets and close to the seismic sources of the mainshocks. The stations recorded strong magnitude events such as the Mw 6.5, as well as hundreds of other aftershocks of magnitude larger than 3.0, in near source region and in peculiar site conditions. Therefore, the data recordings are of broad interest in several research fields and might yield new and important insights on one of the most devastating seismic sequences in Italy.

## Methods

### General information on deployment and seismic stations

The deployment of a temporary network of seismic stations during an emergency is a challenging intervention that involves several steps.

First of all, the logistics in the epicentral area is strongly influenced by the level of damage and by the rescue operations, then the installation has to be done in agreement with the public authorities. For example, the access to the most damaged area at Amatrice was forbidden for several months after the first mainshock in August 2016, then it was not possible to include this zone in the survey.

Second, it is important to individuate sites representative of the geological conditions that can affect the ground motion characteristics, through a rapid and detailed geological survey^10,11^.

Third, the final location of the stations is driven by other minor logistic problems and by the available equipment. For example, indoor stations need to be installed inside buildings that have not been structurally damaged and where the electricity supply is available. However, because the power mains may be off or allowed only for specific purposes after strong earthquakes, another option is to use batteries that can last for few days. In the case of free-field deployment, seismic stations are usually equipped with 12V buffer batteries and solar panels, with the constraint of having favourable sunlight conditions.

Whatever is the type of installation, a GPS antenna has to be placed outdoor with an optimal sky view for having a sufficient number of satellites visible and get an accurate time synchronization and positioning. Finally, the site should also ensure a minimum level of protection against robberies or vandalism.

The full seismic equipment (Supplementary Fig. 2) consists of: (a) a digitizer; (b) one or two 3-component seismic sensors (velocimeter and/or accelerometer); (c) a GPS antenna; (c) 12 V batteries; (d) solar panels and charge regulators (to get a constant voltage as output and charge the batteries, preventing overcharging and protecting them against overvoltage) or an AC-DC converter that is connected to the electrical power mains.

The digitizers are electronic systems that convert the analog signals coming from the sensors (voltage variation) in digital counts, and store the data in local disks (hard disk, solid-state or USB disks, flash or PCMCIA cards) in a proprietary format. Moreover, they represent the unit controls allowing the user to set the recording parameters^20^. Table 1 summarizes the different kind of digitizers used in this study, given the availability of each institution, with some basic specification and the raw binary format of data.

**Table 1:** Characteristics of the used digitizers, including the following data format:

Brand	Model	Dynamic	Number of channels	Raw binary data format
Reftek	R130	24-bit	6	Reftek
Reftek	R130S	24-bit	6	Reftek
Kinemetrics	Quanterra Q330	24-bit	6	MiniSEED
Sara Electronic Instruments	VELBOX	24-bit	3	MiniSEED
Lennartz - Sara Electronic Instruments	Marslite	20-bit	3	MiniSEED
Sara Electronic Instruments	ACEBOX	24-bit	3	MiniSEED
Kinemetrics	ETNA	20-bit	3	EVT
Kinemetrics	K2	20-bit	3	EVT

Reftek (http://www.reftek.com);

MiniSEED (http://ds.iris.edu/ds/nodes/dmc/data/formats/miniseed);

EVT (https://kinemetrics.com).

The seismic sensors are the receivers placed on the ground that measure the incoming seismic wavefield, convert it into electric signal and send it to the recorder through a cable^20^. The most popular kind of sensors used in seismic networks are velocimeters and accelerometers, recording the velocity and the acceleration of the ground motion, respectively. The capacity in recovering the real signal (in velocity or acceleration) as a function of the investigated frequency band is different between the two. In particular, the velocimeters work well above the so-called natural frequency (usually 0.2 Hz, 1 Hz, 2 Hz) and up to 40–50 Hz; outside this frequency range, the signal recovering is poor. Another limitation of the velocimeters is the voltage scale they can support with respect to the real amplitude of the seismic signal: this means that, if the ground motion exceeds a certain threshold, the recorded signal will result saturated.

On the other hand, the accelerometers, especially the new-generation ones, have a flat response in a wider frequency range and are able to record very large amplitudes (usually up to 4 g), but they are very scarce to detect low-amplitude signals.

Therefore, the two kinds of sensors have complementary application: the velocimeters are used to record the low-to-moderate seismic signals, including the seismic ambient noise, while the accelerometers are more suitable for the strong motions. Of course, there is a common range of amplitudes where the two sensors are reliable, and this data duplication can be seen as a backup in case of malfunctioning of one of the two.

The sensors are able to record the ground motion in three different directions, two horizontals (East-West and North-South) and one vertical. This is the reason why the digitizers have 3 or 6 channels (Table 1), indicated as EHE, EHN and EHZ for those dedicated to the velocimeter and HNE, HNN and HNZ for those dedicated to the accelerometer. Usually the sensors are placed directly on the ground, possibly far from buildings to avoid the soil-to-structure interaction^21^ and levelled; better coupling and thermal insulation are obtained digging a hole and covering the sensor with the soil^22^. Even if the seismic equipment is installed inside a building, the sensors should be put outside or at the foundation level, in order to limit the effect of the building vibrations.

Table 2 summarizes the different kind of sensors used in this study, given the availability of each institutions. The Kinemetrics ETNA and K2 as well as the Sara ACEBOX accelerographs have a built-in accelerometer (Episensor for the first two, SA10 for the ACEBOX); Sara VELBOX seismographs have a built-in velocimeter of type SS20. ETNA and K2 and are the only instruments that are not able to record in continuous mode: the acquisition starts only if a given amplitude threshold is exceeded (trigger mode).

**Table 2:** Characteristics of the used 3-component sensors.

Brand	Model	Type	Lower cutoff frequency
Lennartz	Le3d-5s	Velocimeter	0.2 Hz
Kinemetrics	Episensor FBA ES-T	Accelerometer	0 Hz
Sara	SA10	Accelerometer	0 Hz
Sara	SS20	Velocimeter	2 Hz

The installation procedure of each station is a crucial point for obtaining a good data quality^20,23,24^ and then we took much care on this step. In addition, a stand-alone seismic station needs to be periodically checked to control the correct functioning of the apparatus (e.g. if the solar panels are correctly charging the buffer batteries or the data recording failed) and to download the data from the local disks. This maintenance task is particularly important in the very first days of deployment, and every information concerning the station state of health is stored in field sheets. In our case we checked the stations every three days, afterwards we reduced the frequency to one week. The collection of metadata related to the stations (e.g. power settings, location parameters, site name, site code, time and date, checks of working, etc) used during the installation and maintaining phases, have been performed through a self-developed Android mobile app, specifically designed for the campaign operations^25^. By means of this tool, the metadata are automatically converted into our field sheets (Supplementary Fig. 3).

### The experiment

**Fig. 3:**
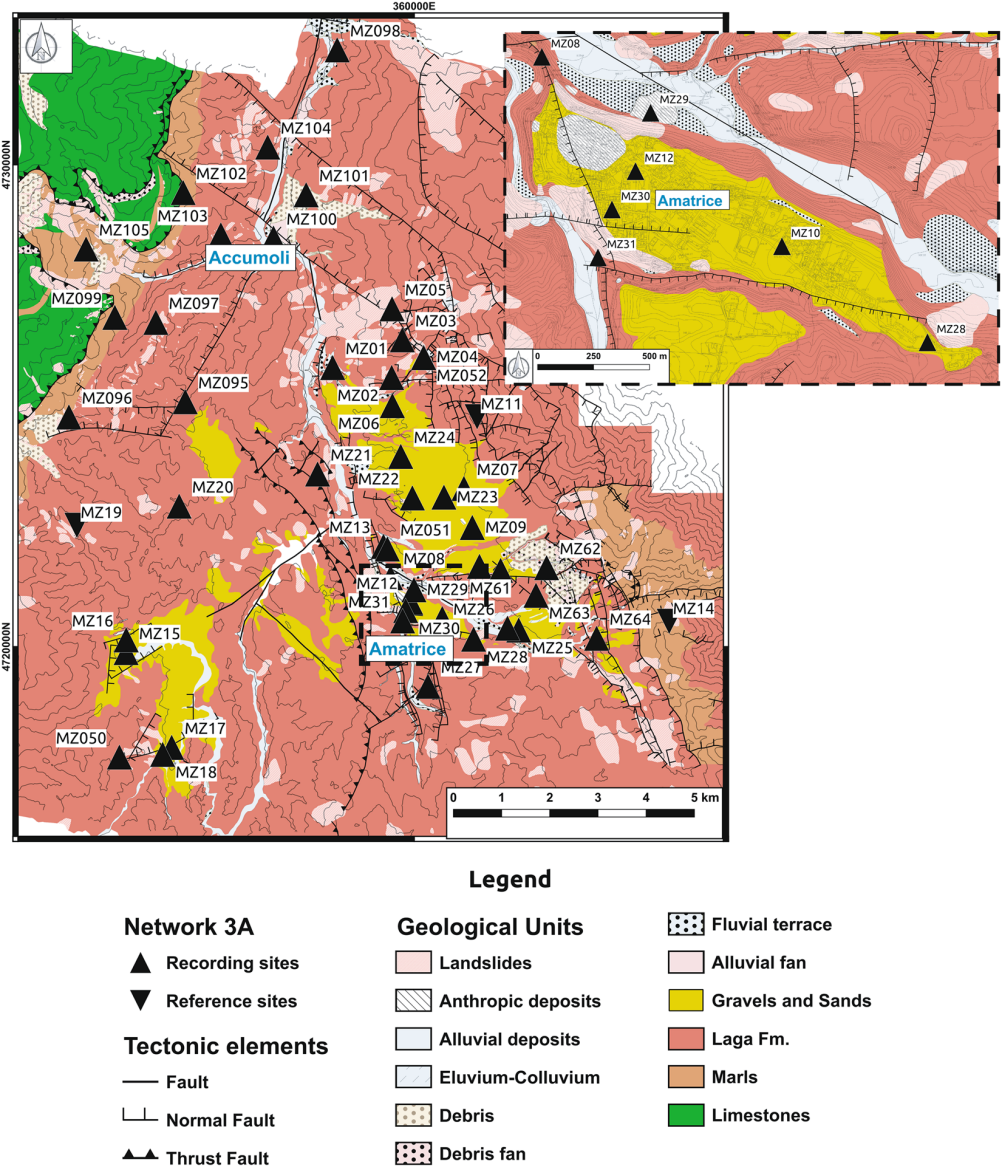
Simplified geological map of the Amatrice and Accumoli municipalities. Position of the seismic stations of the network 3A on a simplified geological map (scale 1:52800); the inlet shows a zoom of the Amatrice village.

The two municipalities of Amatrice and Accumoli, involved in this study, consist of 49 and 16 hamlets on an area of about 175 and 89 square kilometers wide, respectively. Because of the large extension of the area, a total of 29 hamlets was selected for the microzonation studies in the Amatrice and 16 hamlets in Accumoli municipalities, depending on their dimension and on the high level of damage. A preliminary geological survey allowed to identify 50 sites (39 for Amatrice and 11 for Accumoli) representative of the geological condition of the hamlets and of the main villages where to install the seismic stations (Figs 2b and 3); in addition to the stations installed in soils prone to amplification effects, some rock sites have been selected with the aim to use them as reference sites (MZ11, MZ14 and MZ19 in Amatrice municipality). The largest number of stations was installed in the village of Amatrice, due to its size, historical importance and heavy damage (Fig. 3).

The two municipalities lie in the Amatrice Basin, an intrappenninic morpho-structural depression bounded westward by the Sibillini Mts and eastward by the Laga Mts (Fig. 2b). This depression corresponds to a broad N-S-trending syncline, filled by an up to 1.2 km thick sequence of deep-marine siliciclastic turbidites (Laga Fm; Late Miocene)^26,27^. The syncline widens at the footwall of the east-verging Sibillini Mts Thrust front, where it crops out a pelagic carbonate-cherty succession of the Umbria-Marche paleogeographic domain (Jurassic-Middle Miocene), and at the hangingwall block of the seismogenic Mt Gorzano Normal Fault^28^, which exposes uplifted Miocene marls and turbidites (*Marne con Cerrogna* and *Marne a Orbulina* Fms; Laga Fm). Both the turbidites and the carbonate-cherty succession compose the geological bedrock at the basin scale (reddish and green colors in Fig. 3). The bedrock is covered by up to 60 m-thick of Quaternary terrigenous continental deposits, arranged in a complex stack of alluvial fans, fluvial terraces and slope deposits^29,30^. Anthropogenic deposits are found in the hamlets, having thickness rarely exceeding 5–7 m. Geological formations are constrained by both deep^31^ and surface^32–34^ velocity profiles: the surface profiles have been obtained for the microzonation studies performed within the two areas, whereas the deep tomography has been studied in more detail after the Central Italy seismic sequence.

The extensive intervention, also considering the logistic problems during the very first period of the emergency, was possible only with the effective collaboration of the Italian institutions involved in CentroMS and charged to operate in Amatrice and Accumoli municipalities: Istituto Nazionale di Geofisica e Vulcanologia (INGV), Agenzia nazionale per le nuove tecnologie, l’energia e lo sviluppo economico sostenibile (ENEA), Istituto Per La Dinamica Dei Processi Ambientali of Consiglio Nazionale delle Ricerche (CNR-IDPA), Istituto di Metodologie per l’Analisi Ambientale of Consiglio Nazionale delle Ricerche (CNR-IMAA) and Istituto di Geologia Ambientale e Geoingegneria of Consiglio Nazionale delle Ricerche (CNR-IGAG).

Online-only Table 1 summarizes all the information regarding the stations, named with a code consisting of two letters, “MZ”, followed by a two or three digit number.

The stations in Amatrice municipality were installed in free-field using solar panels and buffer batteries, and acquired 100 samples per second (sps) for velocimeters and 200 sps for accelerometers. The ones in Accumoli were placed inside buildings and powered by power mains, recording with a sampling rate of 200 sps. The gain of digitizers was set at the minimum value, to avoid as much as possible the signal saturation for high-magnitude events, especially for velocimeters.

The network operated in continuous recording mode from September 19, 2016, to the end of November 2016, with the exception of the stations in the Accumoli area, which recorded for one month. Some interruption due to powering problems occurred during the experiment but, in general, almost 96% of the whole period has been correctly recorded (Online-only Table 1).

**Fig. 4:**
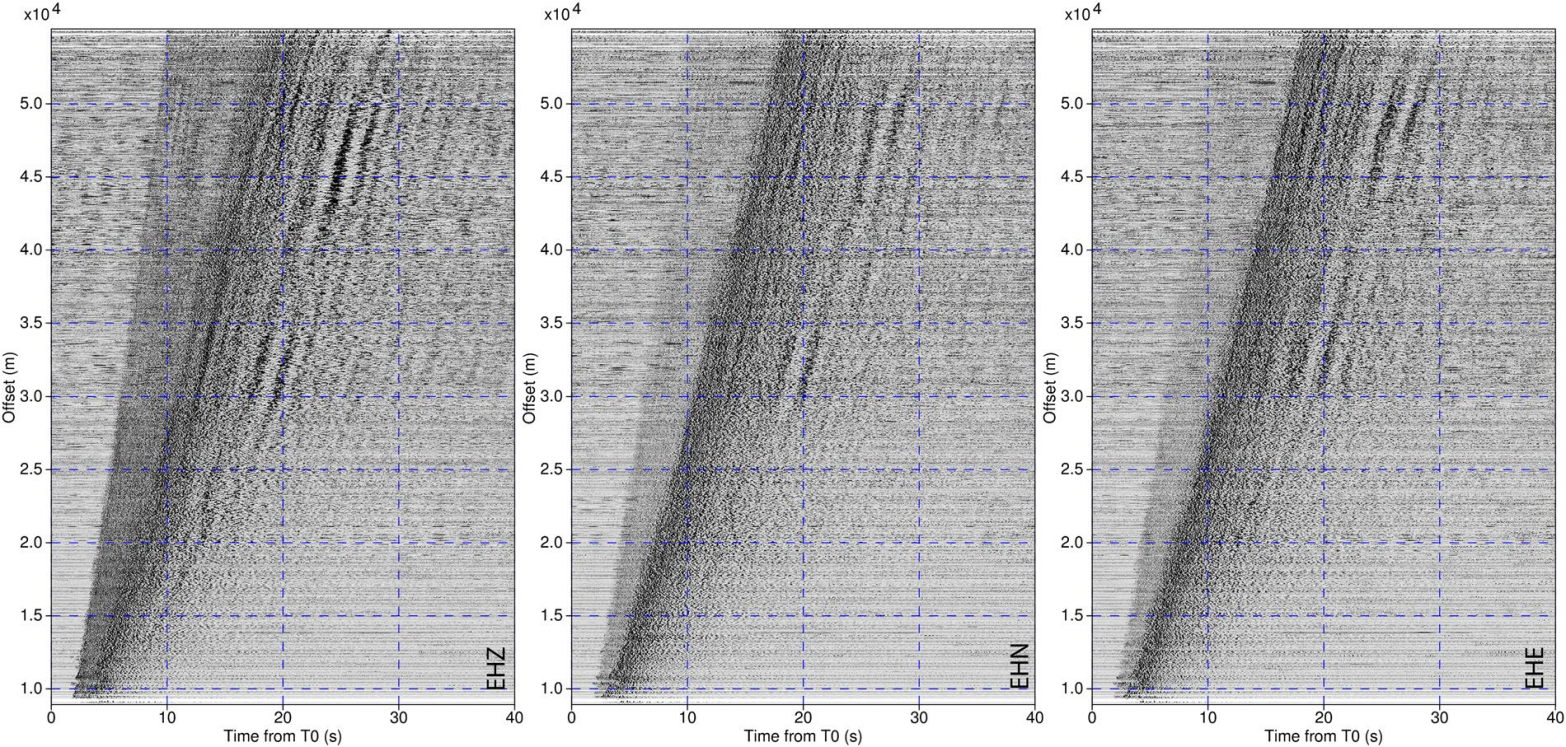
Seismic sections from 45 stations of the network 3A. The sections for the three components of the ground motion are obtained ordering the local events, in the magnitude range 2.5–3.0 recorded by the 45 stations, by the epicentral-station distance. For each section a total number of 1253 events has been considered. Due to this large number, we stacked the normalized waveforms falling in windows of 50 m of distance from the epicenter.

The correct functioning of the stations allowed to record hundreds of earthquakes of the seismic sequence, including the Mw 5.4 and 5.9 on October 26 and the Mw 6.5 occurred close to Norcia on October 30, 2016. Figure 4 represents a seismic section in which each velocimetric recording of local events in the magnitude range 2.5–3 (for a total of 1253 events) is ordered by the epicenter-station distance. In this representation the arrival of P-waves in the vertical components and of S-waves in the horizontal ones is really enhanced.

In addition to those, the network recorded some regional earthquakes and the Mw 8.1 New Zealand event (Table 3).

**Table 3:** Earthquakes of Magnitude larger than 5 recorded by the 3A network; in column 5 “EMSC” stands for Euro Mediterranean Seismological Center.

Time	Latitude	Longitude	Magnitude	Source of localization	Event Location Name
2016-09-23T23:11:20	45.76	26.63	Mw 5.6	SURVEY INGV/EMSC	Romania
2016-09-27T20:57:09	36.3779	27.5647	mb 5.3	SURVEY INGV	Dodecanese Islands, Greece [Sea: Greece]
2016-09-28T07:17:36	36.8174	21.9045	mb 5	SURVEY INGV	Western Peloponnese coast (Greece)
2016-10-15T20:14:49	39.7749	20.6409	mb 5.2	SURVEY INGV	Greece [Land]
2016-10-26T17:10:36	42.8747	13.1243	Mw 5.4	BULLETIN INGV	3 km SW Castelsantangelo sul Nera (MC)
2016-10-26T19:18:05	42.9048	13.0902	Mw 5.9	BULLETIN INGV	3 km S Visso (MC)
2016-10-28T20:02:43	39.2672	13.5468	ML 5.8	BULLETIN INGV	Southern Tyrrhenian e (Sea)
2016-10-30T06:40:17	42.8303	13.1092	Mw 6.5	BULLETIN INGV	5 km NE Norcia (PG)
2016-11-13T11:03:01	−42.5742	172.861	Mwpd 8.1	SURVEY INGV	South Island, New Zealand [Land]

**Fig. 5:**
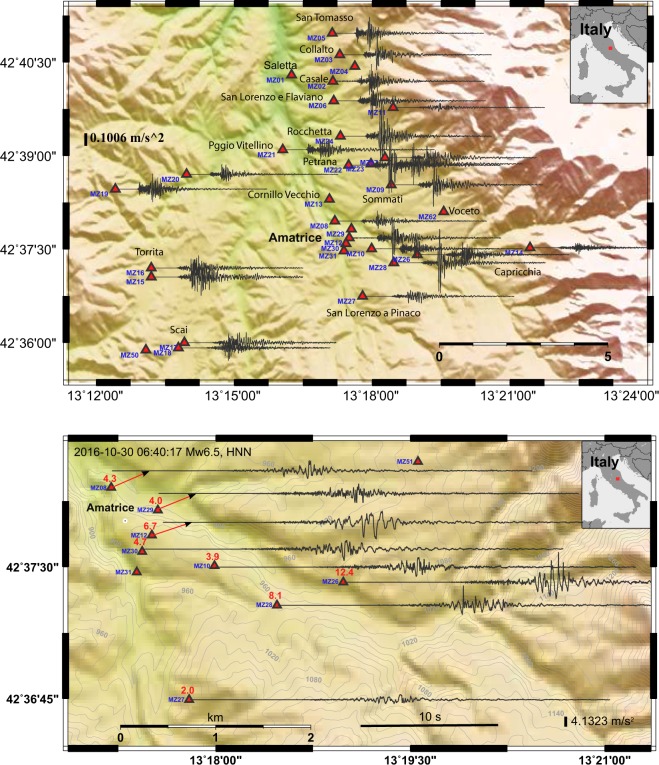
Accelerations of two earthquakes recorded by the network 3A. Top: E-W components of the event occurred on the 16th October 2016 Mw 4.0; Bottom: N-S components recorded by the stations at Amatrice for the strong shock of the 30th of October 2016 (Mw 6.5). The red numbers on the stations location represent the PGA values in m/s^2^ and contour lines represent the ground elevation in meters.

As an example, Fig. 5 shows the HNN component of accelerometric recordings at the Amatrice municipality during the Mw 4 (October 16, 2016 at 09:32) and the recordings in the Amatrice village during the Mw 6.5, the largest mainshock of the Central Italy seismic sequence.

## Data Records

In order to publish the dataset recorded by the stations in Amatrice and Accumoli, we followed several steps:

1. Registration of the temporary seismic network to the *International Federation of Digital Seismograph Networks* (FDSN, https://www.fdsn.org) to assess the uniqueness of the stations ensemble within the worldwide seismic networks; the FDSN code “3A (2016–2016)” was assigned to the network named “Centro di microzonazione sismica Network, 2016 Central Italy seismic sequence (CentroMZ)” (https://www.fdsn.org/networks/detail/3A_2016/).
2. Registration of each station to the *International Station Registry* (ISC, http://www.isc.ac.uk/), giving the assigned name and few information such as the geographic coordinates (e.g. for MZ08 http://www.isc.ac.uk/cgi-bin/stations?stnsearch=STN&sta_list=MZ08).
3. Conversion of the seismic continuous records to the format required by the chosen data repository, the *European Integrated Data Archive* (EIDA; https://www.orfeus-eu.org/data/eida/), a public repository promoted by the *Observatories & Research Facilities for European Seismology* (ORFEUS) non-profit foundation, aimed at providing access to seismic waveform data in European archives.
4. Definition of the agreement between the involved institutions about the data policy.
5. Assignation of a Digital Object Identifier (DOI) to the network 3A.

Regarding the step 3, EIDA hosts the continuous data recording of the main European networks. Since 2013, INGV is the Italian primary node of the EIDA archive (http://eida.rm.ingv.it). Therefore, it was a natural step choosing EIDA also for the network 3A, and using the INGV EIDA node to upload the dataset.

However, in order to upload our dataset to EIDA, some preliminary steps were required:

- Collection of the raw data from the several institutions, with the available metadata information: geographical coordinates, gain, sampling rate, raw format, instrument response
- Conversion of the raw data into the format used in EIDA
- Copy of the data into the EIDA-like structure
- Creation of the metadata volumes for each station.
- Control of the data conversion and the associated metadata volumes.

As shown in Table 1, raw seismic data are saved as binary files in the digitizer’s proprietary format. These data are usually saved in local disks or flash cards that, during the recording time, are periodically substituted to copy data in local storage. The very first step, once collected the whole dataset coming from the field, was then to convert the data into the full SEED format (*Standard for the Exchange of Earthquake Data*), supported by EIDA and maintained by FDSN (http://www.fdsn.org/seed_manual/SEEDManual_V2.4.pdf).

The SEED format is a combination of time series values (binary miniSEED format) along with its metadata (DATALESS). The miniSEED format includes a very limited metadata (their identification and simple state-of-health flags), whereas the DATALESS contain a complete and comprehensive history of metadata for one or many networks and stations (i.e. geographic coordinates and instrument response information often needed to process the time series data).

Supplementary Fig. 4 shows the structure of the archive tree as used in EIDA (SeisComP Data Structure, https://www.seiscomp3.org/doc/applications/slarchive/SDS.html). The folders are organized by year, network code, station codes, and channels. The miniSEED format uses the file name to uniquely identify the time series and provide attribution to the owner of the data. In particular the name must contain: (1) the network code; (2) the station code; (3) the channel code. The miniSEED files are 1-day long, then the name includes also the corresponding Julian date (Supplementary Fig. 4).

The DATALESS volumes are provided separately.

The complete dataset of this study was also uploaded in the *figshare* repository^35^. We maintained the same EIDA-like structure but the repository contains a zip file for each station and a single zip file for the DATALESS files. Moreover, we included a text table containing the relevant information of the local earthquakes (epicentral distance from Amatrice less than 30 km, local or moment magnitude more than 3.0) recorded by the network 3 A during its working period: origin time, epicentral latitude, longitude and depth, local or moment magnitude and the event location name. A complete list of the catalogue can be obtained from the INGV web service (accessible from http://terremoti.ingv.it/en/) using the circular selection centered at Amatrice (Latitude 42.63, Longitude 13.29) as well as the desired radius and magnitude threshold.

### Assigning a DOI and data policy

The data publication required, as final steps, an agreement between the involved institutions about the data policy and the assignment of a regular DOI associated to the network and provided by INGV through a standard procedure. The final DOI of the network 3A was 10.13127/SD/ku7Xm12Yy9.

The data policy has been regulated by a Memorandum of Understanding (MoU) between the involved institutions and the data have been licensed using the Creative Commons License CC BY 4.0. The MoU decided to restrict the data for a given period of time after the publication in EIDA, e.g. 12 months, in order to allow the Authors to use them for the analyses required by the agreement of CentroMS for Civil Protection purposes, and to produce a first publication. During the embargo period, the Authors decided also to make the data available under request for scientific purposes, different from what needed by CentroMS.

The dataset uploaded to EIDA can be requested in two ways:

- using the web interface of EIDA, selecting the network, the stations, the channels and the period of time to download (http://www.orfeus-eu.org/webdc3/)
- using the web services provided for the INGV node (https://www.orfeus-eu.org/data/eida/nodes/INGV/).

A selection of the recorded waveforms, e.g. the accelerometric traces of the strongest earthquakes of the sequence (local magnitude Ml ≥ 4.0), can be also downloaded from the Engineering Strong-Motion Database^36^ (ESM).

The complete dataset can also be downloaded from *figshare*^35^.

## Technical Validation

In order to verify the completeness and correctness of the recorded data, we carried out several checks.

### Completeness of the recording period

**Fig. 6:**
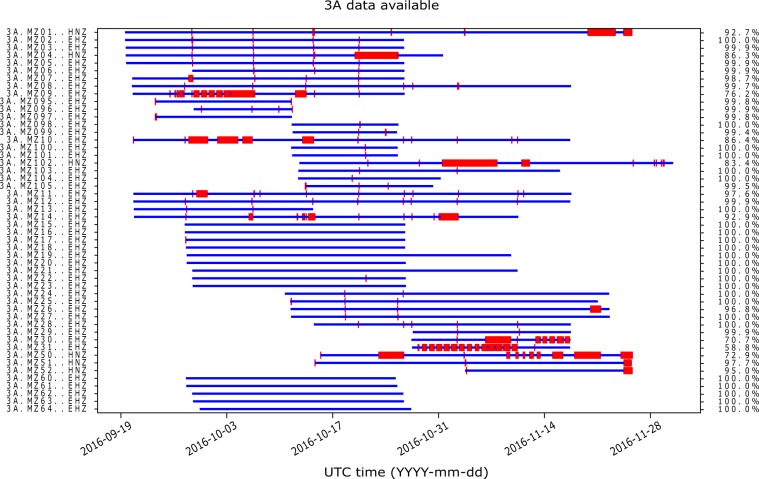
Data availability for the stations of the network 3A. Availability of the recordings for each station as a function of time (blue lines). For clarity only the vertical components have been used. Gaps in the recordings (red) are mainly due to days with weak solar radiation. The figure has been realized using the ***obspy***-***scan*** tool included in the ObsPy package (https://docs.obspy.org/packages/autogen/obspy.imaging.scripts.scan.html).

For each station operating in continuous recording mode, we detected the ratio between the effective operating time with respect to the total lifetime. The actual uptimes, calculated with the program ***msi*** (miniSEED inspector, https://github.com/iris-edu/msi), are detailed in Online-only Table 1. Figure 6 shows the graphs of data availability for all the stations. Despite the problems occurred to the station MZ31 (a charge regulator not working properly) and a few others, the result in terms of correct operation was satisfactory, since stations on average worked properly for about 95% of the life time.

### Data integrity

The data integrity of miniSEED files is a kind of internal consistency of data, and any discrepancies indicate that the contents of the data can be garbled (http://www.fdsn.org/seed_manual/SEEDManual_V2.4.pdf). We verified this feature on the whole miniSEED archive using the program ***qmerge*** (http://www.ncedc.org/qug/software/ucb/), and we found that a small number of files recorded from one of the stations did not meet the requirement, due to a malfunction probably caused by water infiltration; we discarded these files from the final archive.

### Temporal variation of the noise level

We estimated the power spectral density (PSD) of the noise at the various stations and compared it to the standard low and high noise models (NLNM and NLHM)^37^. In general this method is used to evaluate the noise level in sites that are expected to be characterized by low seismic noise and are far from anthropic noise sources; in our case, the PSD could be useful for detecting possible operating problems and looking at the temporal variation of the noise level.

The PSD was obtained using a self-made procedure based on the PPSD class of the ***obspy*** package

(https://docs.obspy.org/packages/autogen/obspy.signal.spectral_estimation.PPSD.html#obspy.signal.spectral_estimation.PPSD), in which calculations are based on the routine used by McNamara *et al*.^38^.

Supplementary Fig. 5 shows the PSD of stations that were operating on September 26, 2016 for the vertical velocimetric component of motion (channel EHZ) evaluated on a 24-hour length recording. It is worth noting that in the high frequency range the curves sometimes go slightly out of the range defined by NLNM and NHNM models; this is probably due to the high seismicity and anthropic noise sources of the area in the period of the experiment. Moreover, some stations are not located on a rock site, then they could be affected by amplification effects.

### Check of the instrumental response

For each station equipped with both accelerometer and velocimeter we compared the recordings of several earthquakes from the two sensors, after calculating the derivatives of the signals coming from the velocimeter. Although the sensors are not obviously installed exactly in the same position, there are always slight orientation errors, and the two types of instruments do not have the same accuracy, we expect the trend of the waveforms to be substantially the same, at least in the common working frequency range of the two sensors. Any discrepancies are probably due to the malfunctioning of some components of the station, or to some mismatch in the metadata used to convert waveforms into physical units.

**Fig. 7:**
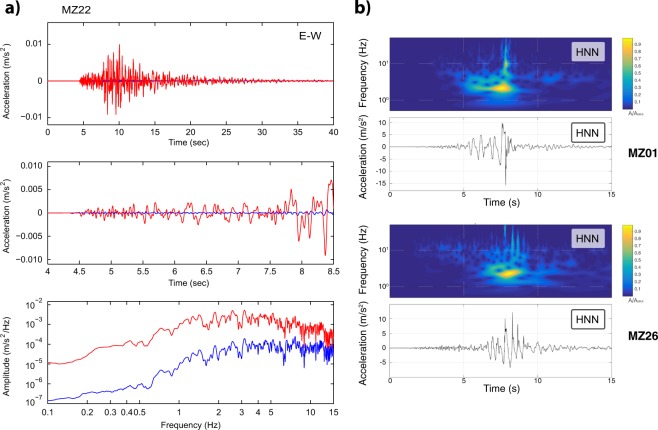
Anomalies on the recordings. (**a**) Comparison between the E-W recordings from accelerometer (red curves) and velocimeter (blue curves) at station MZ22 of the ML 3.2 event (October 2, 2016, 8 km west of Accumoli). Top and middle panels show the entire overlaid signals and limited to few seconds including the P- and S- arrivals, respectively; the bottom panel show the Fourier amplitude spectra between 0.1 and 15 Hz. (**b**) Accelerograms and relative S-transform of the 30th of October Mw 6.5 earthquake recorded at station MZ01 (top panels) and at station MZ26 (bottom panels).

Figure 7a shows the comparison between the E-W accelerometric and velocimetric recordings of the ML 3.2 earthquake occurred on October 2, 2016, for the MZ22 station (see Online-only Table 1). It is clear that the derivative of EHE component of the velocimeter recorded much lower amplitudes than the corresponding HNE component of the accelerometer. We verified that this behaviour was due to a malfunction of the sensor and consequently we decided to exclude the EHE recordings taken in the period of malfunctioning from the final dataset.

### Anomalies observed for large shaking

Because of the importance of the 30th of October Mw 6.5 earthquake, the recordings of this event have been accurately checked. Comparing the accelerometric recordings, five stations show a clear different behaviour: MZ01, MZ12, MZ21, MZ26 and MZ28. In particular, the accelerogram of MZ01 station contains an anomalous peak of about 1.5g on the HNN component (Fig. 7b, top panels); the S-transform shows that this peak has a high frequency content, still evident on the velocity and displacement of the ground motion, obtained through integration and double integration of the accelerometric records, respectively. We investigated if this peak was present at the closest station MZ04, located at about 1.9 km far from MZ01: the absence of this peak at MZ04, that on the contrary shows a maximum acceleration of 0.6 g along the HNN component, is a good reason to believe that the cause of the anomalous peak is not physical, but more likely related to the missing fix of the accelerometer to the ground that made the sensor jump during the strong shaking.

The Mw 6.5 earthquake recordings at MZ12 station present a clear clipping effect due to the low full-scale range of the accelerometer (±0.5 g). Finally, the accelerograms of MZ21, MZ26 and MZ28 stations present different anomalous spikes during the strong motion. These spikes are present in all the three components and in a large range of frequencies (Fig. 7b, bottom panels), and they seem to be superimposed on the correct recorded waveform. At the moment we don’t have exhaustive explanations for this behaviour, but it surely deserves deep investigation because it can have several causes, from a simple electronic problem to a temporary decoupling between sensors and ground or to nonlinear soil behaviour during the strong motion. Consequently, the accelerograms of the Mw 6.5 earthquake recorded at stations MZ01, MZ12, MZ21, MZ26 and MZ28 have been clearly labelled into EIDA and ESM database, in order to avoid their incorrect use.

### Instrumental response at low frequency and time synchronization

A teleseism is by definition an earthquake occurred at a great distance (at least 2000 km) from the stations from which it is registered. The propagation medium attenuates high frequency components with increasing distance, then a teleseismic recording is very rich in energy at low frequencies (f < 1 Hz). If the site has amplification effects in this frequency range, the teleseismic recordings can be very useful in the definition of these effects.

On the other hand, if we have several stations in a limited area that do not show amplification effects at low frequency, the teleseism will travel from the distant source to this area, likely seeing it as a single point, e.g. all the measurement points will record the teleseismic event with the same amplitude; moreover, since the incidence is almost vertical, the arrival times at the various stations are expected to be substantially the same. This is particularly evident if the wavelengths of the incident wavefield are higher than the size of the area.

Because all stations of the network 3A do not have amplification effects at frequency lower than 1 Hz^19^, the comparison of teleseismic events recorded simultaneously by the stations is a good way for checking both their correct working in the low-frequency range (the waveforms are expected to show similar amplitudes), and the correct synchronization.

**Fig. 8:**
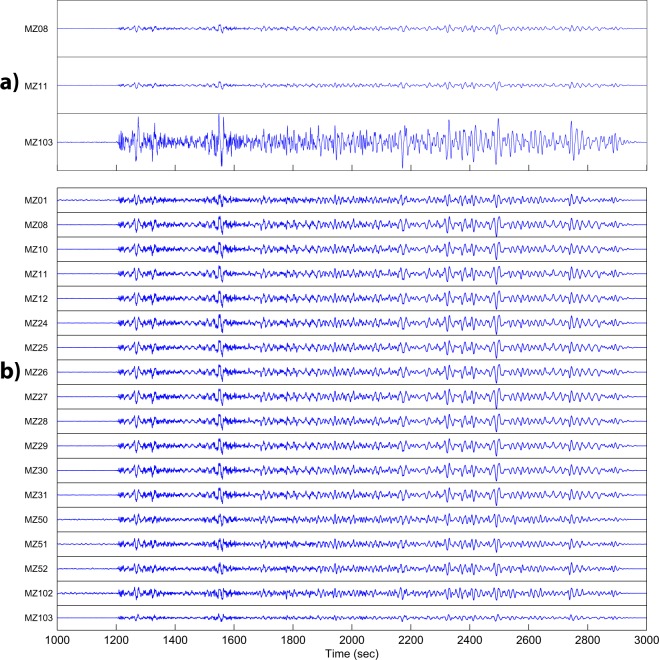
Vertical components recorded by the network 3A during the November 13, 2016, Mw 8.1 New Zealand earthquake: (**a**) Recordings at stations MZ08, MZ11, MZ103, showing the anomalous amplitude of MZ103; (**b**) Vertical components at all stations after correction of the metadata associated to station MZ103. Waveforms are normalized with respect to the maximum amplitude, so that the values on the y axis are in the range [−1 1]; time is relative to the origin time of the earthquake.

We made this comparison using the recordings of the Mw 8.1 earthquake occurred in New Zealand on November 13, 2016. Since the sensors used in the experiment have different characteristics, we previously corrected all the recordings for the instrumental response, and filtered below 1 Hz to further reduce the high frequency content of the signals. The result is summarized in Fig. 8. From this comparison, the time synchronization among stations is verified but amplitudes recorded by station MZ103 strongly differs from the expected trend (see top panel of Fig. 8, comparing with MZ08 and MZ11 which are used as reference because similar to all the others). This led us to re-verify the instrumental characteristics of all the stations, and the final result is shown in the bottom panel of Fig. 8. Although we are sure that the instrumental responses were correct, station MZ103 still shows amplitudes much different from those of the other stations. This problem has been observed also for other three stations (namely MZ096, MZ097, MZ105). Therefore, we decided to maintain the data of these stations on the EIDA archive, adding a warning that they are not fully reliable in the low frequency range (less than 1 Hz).

### Example of data usage

The technical validation allowed us to obtain a reliable and homogenous dataset of continuous waveforms and associated metadata. They can be used for different purposes, ranging from information and recommendations for the civil protection to fundamental seismological research on earthquake generation and wave propagation.

Since the early data collection, earthquake and noise recordings have been used for seismic microzonation studies of level 3^13^ to provide useful information for the management of the early post-emergency phase in the 142 damaged municipalities identified by the Italian Civil Protection. In particular, they have been used by Priolo *et al*.^19^ as representative of the site response for each locality (by providing several site parameters, such as the resonance frequency, amplification factors and empirical transfer functions) and to identify possible reference sites, i.e., a station installed on outcropping rock with a flat site response^39^. In the same framework, the largest magnitude earthquakes have been used by Pagliaroli *et al*.^16^ as input motion, compatible with the Uniform Hazard Spectrum at rock conditions as proposed by the Italian Building Code^40^, for the execution of 1D-2D numerical analysis using equivalent-linear visco-elastic approaches.

The geological and geophysical data together with the collected dataset of seismic signals were also exploited to investigate the site response at some sites that experienced high level of damage (Imcs > IX)^33^, finding often a good relationship with the levels of amplification.

The site response was also evaluated in the Amatrice village^32^, where spatial variation of amplification factors suggests a lateral variability in the geological conditions of the sedimentary layers and in the impedance contrast. The simulation of the ground motion of the Mw 6.0 August 24, 2016, earthquake at sites in the downtown, obtained by means of the recordings of the network 3A, can be a useful tool to exploit the damage distribution (A. Todrani, personal communication).

The dataset has been also used for other purposes, such as the computation of earthquake parameters for the events recorded by the Italian Seismic Network^41^ and the test of new methods to achieve a significant improvement in the solution accuracy and magnitude completeness^42^: aftershock distribution and relocated mainshocks give insight into the complex architecture of major causative and subsidiary faults, thus providing crucial constraints on multi-segment rupture models. Kinematic and dynamic source modeling of the strongest shocks, together with the study of the S-wave polarization (E. Tinti, personal communication) and of peculiar wave propagation (D. Famiani, personal communication), can largely benefit of the network 3A data.

## Supplementary Information

Supplementary Information

## Data Availability

Softwares used to store miniSEED files in EIDA and to create the DATALESS volumes are only partially established in the seismological community. For the purpose of storing in EIDA structure, we used a home-made procedure (written as bash shell script) based on the program ***qmerge*** (http://www.ncedc.org/qug/software/ucb/). The procedure repackages the miniSEED files coming from the digitizers (or after a conversion from the raw proprietary format) into daily files organized as explained above (Supplementary Fig. 4), and it allows the user to update the miniSEED file header according to the parameters defined in the configuration file (e.g, the network and station code, the channel fields); this is necessary because some digitizers do not set automatically the values of these fields. The Incorporated Research Institutions for Seismology (IRIS, https://www.iris.edu/hq/) provides the graphical program ***pdcc*** (https://ds.iris.edu/ds/nodes/dmc/software/downloads/pdcc/) to create the DATALESS files. This code allows to view, create and edit SEED metadata files by means of handy tools. The catalog from which the program retrieves the data concerning the instrument responses is the Nominal Response Library (NRL) maintained by IRIS^43^, whereas the coordinates, description and all the information regarding the stations must be entered by the user in appropriate text fields. Nevertheless, because the seismic instrumentation used by the network 3A is not entirely present in the IRIS NRL or some specific information have to be retrieved from the datasheets of the manufacturers, we prefer to use a home-made procedure (written as bash shell script) to create the DATALESS files. The use of a command-line code was very convenient, given the large number of stations. The procedure retrieves the information about the instrumentation (sensor responses given in terms of poles and zeros, filters of the digitizers, and so on) from text files organized in a kind of local catalog, and the parameters of the stations (coordinates, start and end dates, etc.) from a database that collects them. However, ***pdcc*** was useful for the technical validation of the obtained DATALESS files.

## Online-only Table

**Online-only Table 1:** Information regarding the stations.

Station code	Location	Latitude	Longitude	Elevation	Data availability (%)	Start-date	End-date	Digitizer type	Sensor type	Full scale range	Samples per second	Institution
MZ01	Saletta	42.67158	13.27081	859	92.7	2016-09-19	2016-10-14	REFTEK130	LE3D-5S	0.0125 m/s	100	INGV
						2016-09-19	2016-10-14	REFTEK130	EPISENSOR-FBA-ES-T	2 g	200	INGV
						2016-10-14	2016-11-25	ACEBOX	SA10	1 g	200	CNR-IMAA
MZ02	Casale	42.66995	13.28581	963	100	2016-09-19	2016-10-26	REFTEK130	LE3D-5S	0.0125 m/s	100	INGV
						2016-09-19	2016-10-26	REFTEK130	EPISENSOR-FBA-ES-T	0.5 g	200	INGV
MZ03	Collalto	42.67702	13.28832	1093	99.9	2016-09-19	2016-10-26	REFTEK130	LE3D-5S	0.0125 m/s	100	INGV
						2016-09-19	2016-10-26	REFTEK130	EPISENSOR-FBA-ES-T	2 g	200	INGV
MZ04	Cossito	42.67390	13.29381	978	86.3	2016-09-19	2016-10-14	REFTEK130	LE3D-5S	0.0125 m/s	100	INGV
						2016-09-19	2016-10-14	REFTEK130	EPISENSOR-FBA-ES-T	0.5 g	200	INGV
						2016-10-14	2016-11-02	ACEBOX	SA10	1 g	200	CNR-IMAA
MZ05	San Tomasso	42.68277	13.28556	1062	99.9	2016-09-19	2016-10-26	REFTEK130	LE3D-5S	0.0125 m/s	100	INGV
						2016-09-19	2016-10-26	REFTEK130	EPISENSOR-FBA-ES-T	0.5 g	200	INGV
MZ06	San Lorenzo Flaviano	42.66469	13.28614	975	99.9	2016-09-19	2016-10-26	REFTEK130	LE3D-5S	0.0125 m/s	100	INGV
						2016-09-19	2016-10-26	REFTEK130	EPISENSOR-FBA-ES-T	0.5 g	200	INGV
MZ07	Sant’Angelo	42.64949	13.30468	1012	98.7	2016-09-19	2016-10-26	REFTEK130	LE3D-5S	0.0125 m/s	100	INGV
						2016-09-19	2016-10-26	REFTEK130	EPISENSOR-FBA-ES-T	0.5 g	200	INGV
MZ08	Amatrice RAN	42.63254	13.28656	897	99.7	2016-09-20	2016-11-18	REFTEK130	LE3D-5S	0.0125 m/s	100	INGV
						2016-09-20	2016-11-18	REFTEK130	EPISENSOR-FBA-ES-T	0.5 g	200	INGV
MZ09	Sommati	42.64223	13.30699	990	76.2	2016-09-20	2016-10-26	REFTEK130	LE3D-5S	0.0125 m/s	100	INGV
						2016-09-20	2016-10-26	REFTEK130	EPISENSOR-FBA-ES-T	0.5 g	200	INGV
MZ10	Amatrice - San Cipriano	42.62512	13.29985	979	86.4	2016-09-20	2016-11-18	REFTEK130	LE3D-5S	0.0125 m/s	100	INGV
						2016-09-27	2016-11-18	REFTEK130	EPISENSOR-FBA-ES-T	1 g	200	INGV
MZ11	bedrock nord Sant’Angelo	42.66291	13.30765	1231	97.6	2016-09-20	2016-11-18	REFTEK130	LE3D-5S	0.0125 m/s	100	INGV
						2016-09-20	2016-11-18	REFTEK130	EPISENSOR-FBA-ES-T	2 g	200	INGV
MZ12	Amatrice giardino	42.62802	13.29179	958	99.9	2016-09-20	2016-11-18	REFTEK130	LE3D-5S	0.0125 m/s	100	INGV
						2016-09-20	2016-11-18	REFTEK130	EPISENSOR-FBA-ES-T	0.5 g	200	INGV
MZ13	Cornillo Vecchio	42.63838	13.28460	904	100	2016-09-20	2016-10-14	REFTEK130	LE3D-5S	0.0125 m/s	100	INGV
						2016-09-20	2016-10-14	REFTEK130	EPISENSOR-FBA-ES-T	1 g	200	INGV
MZ14	Capricchia bedrock	42.62540	13.35730	1391	92.9	2016-09-20	2016-11-11	REFTEK130	LE3D-5S	0.0125 m/s	100	INGV
						2016-09-20	2016-11-11	REFTEK130	EPISENSOR-FBA-ES-T	0.5 g	200	INGV
MZ15	Torrita terrazzo basso	42.61754	13.22004	1083	100	2016-09-27	2016-10-27	Q330	LE3D-5S	0.0175 m/s	100	INGV
						2016-09-27	2016-10-27	Q330	EPISENSOR-FBA-ES-T	2 g	200	INGV
MZ16	Torrita terrazzo alto	42.61992	13.22000	1003	100	2016-09-27	2016-10-27	Q330	LE3D-5S	0.0175 m/s	100	INGV
						2016-09-27	2016-10-27	Q330	EPISENSOR-FBA-ES-T	2 g	200	INGV
MZ17	Scai Agriturismo Narcisi	42.60002	13.23207	987	100	2016-09-27	2016-10-27	Q330	LE3D-5S	0.0175 m/s	100	INGV
						2016-09-27	2016-10-27	Q330	EPISENSOR-FBA-ES-T	2 g	200	INGV
MZ18	Scai alta	42.59867	13.22982	1001	100	2016-09-27	2016-10-27	Q330	LE3D-5S	0.0175 m/s	100	INGV
						2016-09-27	2016-10-27	Q330	EPISENSOR-FBA-ES-T	2 g	200	INGV
MZ19	Pasciano cimitero	42.64109	13.20692	1107	100	2016-09-27	2016-11-10	Q330	LE3D-5S	0.0175 m/s	100	INGV
						2016-09-27	2016-11-10	Q330	EPISENSOR-FBA-ES-T	2 g	200	INGV
MZ20	Collemoresco	42.64510	13.23269	1010	100	2016-09-27	2016-10-27	Q330	LE3D-5S	0.0175 m/s	100	INGV
						2016-09-27	2016-10-27	Q330	EPISENSOR-FBA-ES-T	2 g	200	INGV
MZ21	Poggio Vitellino	42.65163	13.26755	1046	100	2016-09-28	2016-11-11	Q330	LE3D-5S	0.0175 m/s	100	INGV
						2016-09-28	2016-11-11	Q330	EPISENSOR-FBA-ES-T	1 g	200	INGV
MZ22	Petrana	42.64753	13.29168	1015	73.3	2016-09-28	2016-10-27	Q330	LE3D-5S	0.0175 m/s	100	INGV
						2016-09-28	2016-10-27	Q330	EPISENSOR-FBA-ES-T	2 g	200	INGV
MZ23	Faizzone	42.64779	13.29965	1025	100	2016-09-28	2016-10-27	Q330	LE3D-5S	0.0175 m/s	100	INGV
						2016-09-28	2016-10-27	Q330	EPISENSOR-FBA-ES-T	2 g	200	INGV
MZ24	Rocchetta	42.65524	13.28852	912	100	2016-10-10	2016-11-22	REFTEK130	LE3D-5S	0.0125 m/s	100	INGV
						2016-10-10	2016-11-22	REFTEK130	EPISENSOR-FBA-ES-T	1 g	100	INGV
MZ25	Retrosi 1	42.62325	13.31925	1009	100	2016-10-11	2016-11-21	REFTEK130	LE3D-5S	0.0125 m/s	100	INGV
MZ26	Retrosi 2	42.62356	13.31640	983	96.8	2016-10-11	2016-11-22	REFTEK130	LE3D-5S	0.0125 m/s	100	INGV
						2016-10-11	2016-11-22	REFTEK130	EPISENSOR-FBA-ES-T	1 g	100	INGV
MZ27	San Lorenzo a Pinaco	42.61241	13.29658	1007	100	2016-10-11	2016-11-22	REFTEK130	LE3D-5S	0.0125 m/s	100	INGV
						2016-10-11	2016-11-22	REFTEK130	EPISENSOR-FBA-ES-T	1 g	100	INGV
MZ28	Amatrice agriturismo	42.62138	13.30788	992	100	2016-10-14	2016-11-18	REFTEK130	LE3D-5S	0.0125 m/s	100	INGV
						2016-10-14	2016-11-18	REFTEK130	EPISENSOR-FBA-ES-T	1 g	200	INGV
MZ29	Amatrice Caseificio storico	42.63040	13.29250	881	99.9	2016-10-27	2016-11-18	REFTEK130	LE3D-5S	0.0125 m/s	100	INGV
						2016-10-27	2016-11-18	REFTEK130	EPISENSOR-FBA-ES-T	0.5 g	200	INGV
MZ30	Amatrice Alberghiero	42.62643	13.29058	951	70.7	2016-10-27	2016-11-18	REFTEK130	LE3D-5S	0.0125 m/s	100	INGV
						2016-10-27	2016-11-18	REFTEK130	EPISENSOR-FBA-ES-T	0.5 g	200	INGV
MZ31	Amatrice Fosso in Flysch	42.62455	13.28988	878	58.8	2016-10-27	2016-11-18	REFTEK130	LE3D-5S	0.0125 m/s	100	INGV
						2016-10-27	2016-11-18	REFTEK130	EPISENSOR-FBA-ES-T	0.5 g	200	INGV
MZ50	Pozzo Varoni	42.59800	13.21800	1161	72.9	2016-10-15	2016-11-25	ACEBOX	SA10	1 g	200	CNR-IMAA
MZ51	Cornillo Vecchio	42.63795	13.28558	902	97.7	2016-10-14	2016-11-25	ACEBOX	SA10	1 g	200	CNR-IMAA
MZ52	Cossito	42.67378	13.29381	971	95	2016-11-02	2016-11-25	ACEBOX	SA10	1 g	200	CNR-IMAA
MZ60	Cascello	42.63500	13.30900	982	100	2016-09-27	2016-10-25	REFTEK130S	LE3D-5S	0.0175 m/s	100	IDPA-CNR
MZ61	Collepagliuca	42.63432	13.31438	1001	100	2016-09-27	2016-10-25	REFTEK130S	LE3D-5S	0.0175 m/s	100	IDPA-CNR
						2016-10-24	2016-11-24	ETNA	EPISENSOR-FBA-ES-T	0.5 g	200	CNR-IMAA
MZ62	Voceto	42.63500	13.32600	1086	100	2016-09-28	2016-10-26	REFTEK130S	LE3D-5S	0.0175 m/s	100	IDPA-CNR
MZ63	Collecreta	42.62981	13.32345	1071	100	2016-09-28	2016-10-26	REFTEK130S	LE3D-5S	0.0175 m/s	100	IDPA-CNR
						2016-10-15	2016-11-24	K2	EPISENSOR-FBA-ES-T	0.5 g	200	CNR-IMAA
MZ64	Capricchia	42.62200	13.33900	1111	100	2016-09-29	2016-10-27	REFTEK130S	LE3D-5S	0.0175 m/s	100	IDPA-CNR
MZ095	Colle Spada	42.66462	13.23369	1036	99.8	2016-09-23	2016-10-11	VELBOX	SS20	0.005 m/s	200	ENEA
MZ096	Colle Posta	42.66131	13.20430	1073	99.9	2016-09-23	2016-10-11	VELBOX	SS20	0.005 m/s	200	ENEA
MZ097	Cassino	42.67932	13.22582	1079	99.8	2016-09-23	2016-10-11	VELBOX	SS20	0.005 m/s	200	ENEA
MZ098	Grisciano 1	42.73093	13.27031	700	100	2016-10-11	2016-10-25	MARS UPGRADED	LE3D-5S	0,000655 m/s	200	ENEA
MZ099	Terracino	42.68010	13.21540	1151	99.4	2016-10-11	2016-10-25	MARS UPGRADED	LE3D-5S	0,000655 m/s	200	ENEA
MZ100	Fonte del Campo	42.69553	13.25516	737	100	2016-10-11	2016-10-25	MARS UPGRADED	LE3D-5S	0,000164 m/s	200	ENEA
MZ101	Illica	42.70374	13.26322	849	100	2016-10-11	2016-10-25	MARS UPGRADED	LE3D-5S	0,000164 m/s	200	ENEA
MZ102	Accumoli Madonna delle Coste	42.70367	13.23205	1055	83.4	2016-10-12	2016-11-30	ACEBOX	SA10	2 g	200	ENEA
MZ103	Accumoli campo sportivo	42.69592	13.24181	875	100	2016-10-12	2016-11-16	VELBOX	SS20	0.005 m/s	200	ENEA
MZ104	Tino	42.71250	13.25322	838	100	2016-10-12	2016-10-31	VELBOX	SS20	0.005 m/s	200	ENEA
MZ105	San Giovanni	42.69275	13.20776	1010	99.5	2016-10-13	2016-10-30	VELBOX	SS20	0.005 m/s	200	ENEA

The main info are: name, place, coordinates, start and end recording time and type of seismic instruments. The value in column “full scale range” takes into account the full scale range of both the sensor and digitizer.
