# Supplementary material for: Temporary dense seismic network during the 2016 Central Italy seismic emergency for microzonation studies
Source: Sci Data. 2019 Sep 25;6:182. doi: 10.1038/s41597-019-0188-1 (PMC6761093; doi:10.1038/s41597-019-0188-1)
Supplement: Supplementary file 1 — Supplementary Information [file 41597_2019_188_MOESM1_ESM.pdf]

Supplementary Information

Title

Temporary dense seismic network during the 2016 Central Italy seismic emergency for microzonation studies

Authors

Fabrizio Cara et al.

Contents:

Page 2: Supplementary Figure 1. Example of standard technical sheet for a station of the network 3A.

Page 3: Supplementary Figure 2. Example of deployment of a seismic station in free-field.

Page 4: Supplementary Figure 3. Example of field sheet for station MZ05.

Page 5: Supplementary Figure 4. Schematic view of the EIDA Archive structure.

Page 6: Supplementary Figure 5. PSD on seismic noise.

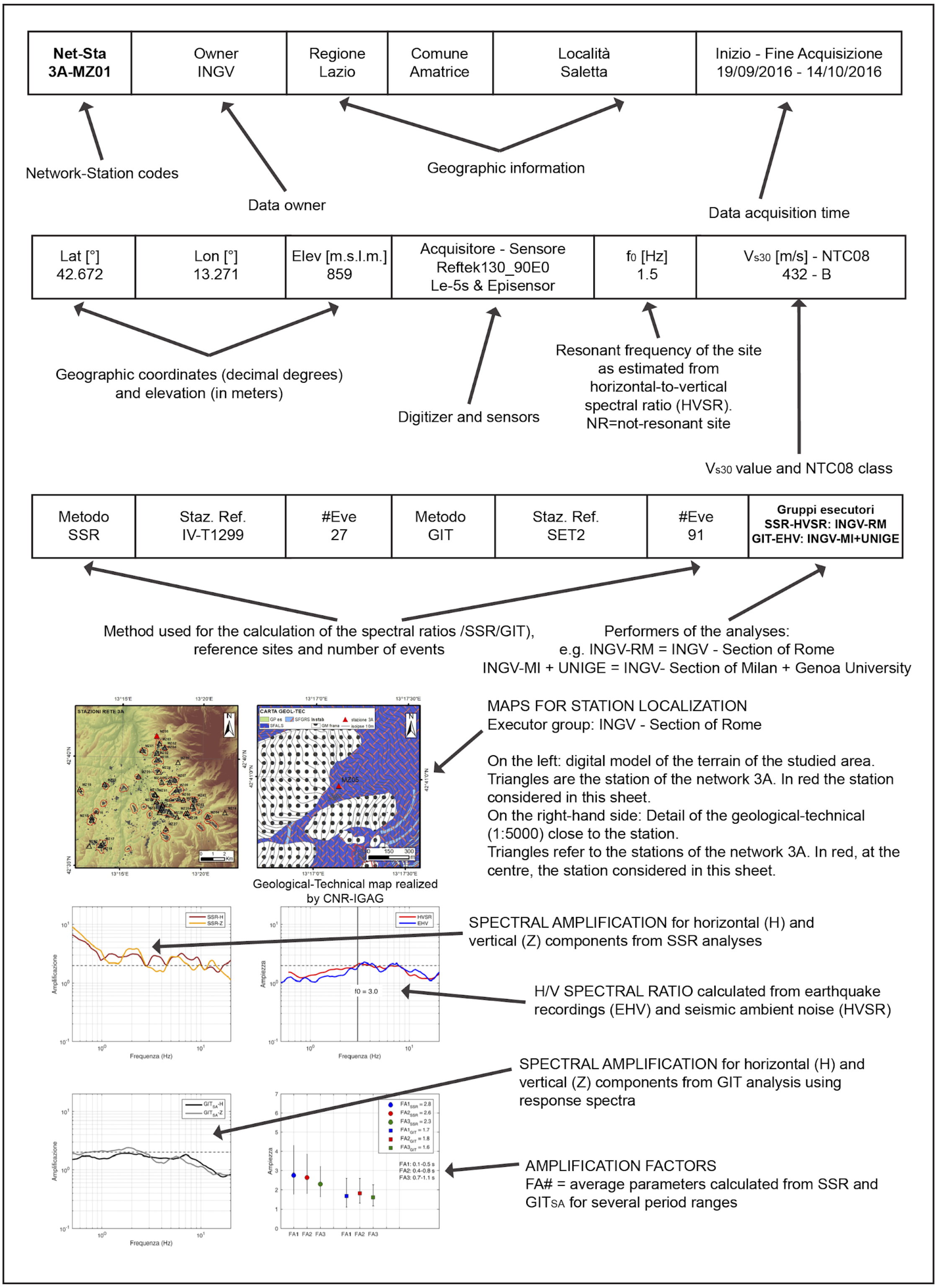

Supplementary Figure 1. Example of standard technical sheet for a station of the network 3A.

The sheet includes the analyses on the recorded data[19,33]: standard spectral ratios (SSR), horizontal-to-vertical (H/V) spectral ratios both on earthquakes and noise, spectral amplification with the generalized inversion technique (GIT), amplification factors (FA).

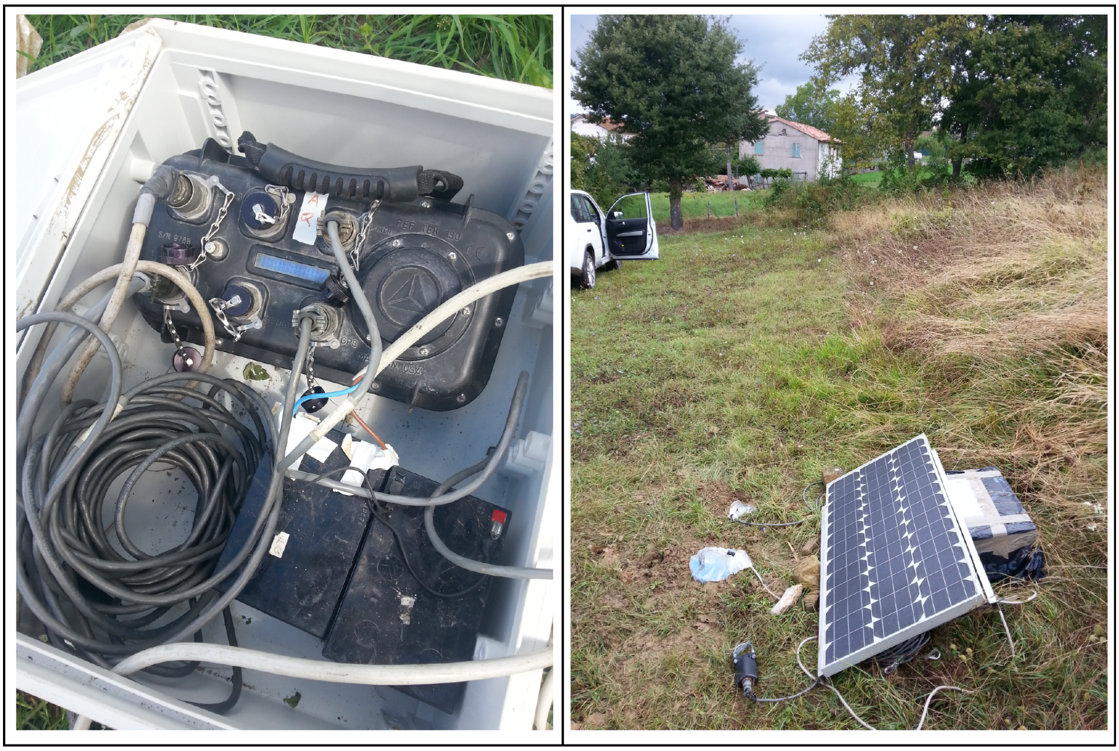

Supplementary Figure 2. Example of a seismic station deployment in free-field.

Left: box with the digitizer (Reftek R130) and batteries.

Right: solar panel, GPS antenna and buried sensors (Lennartz Le3d-5s and Episensor).

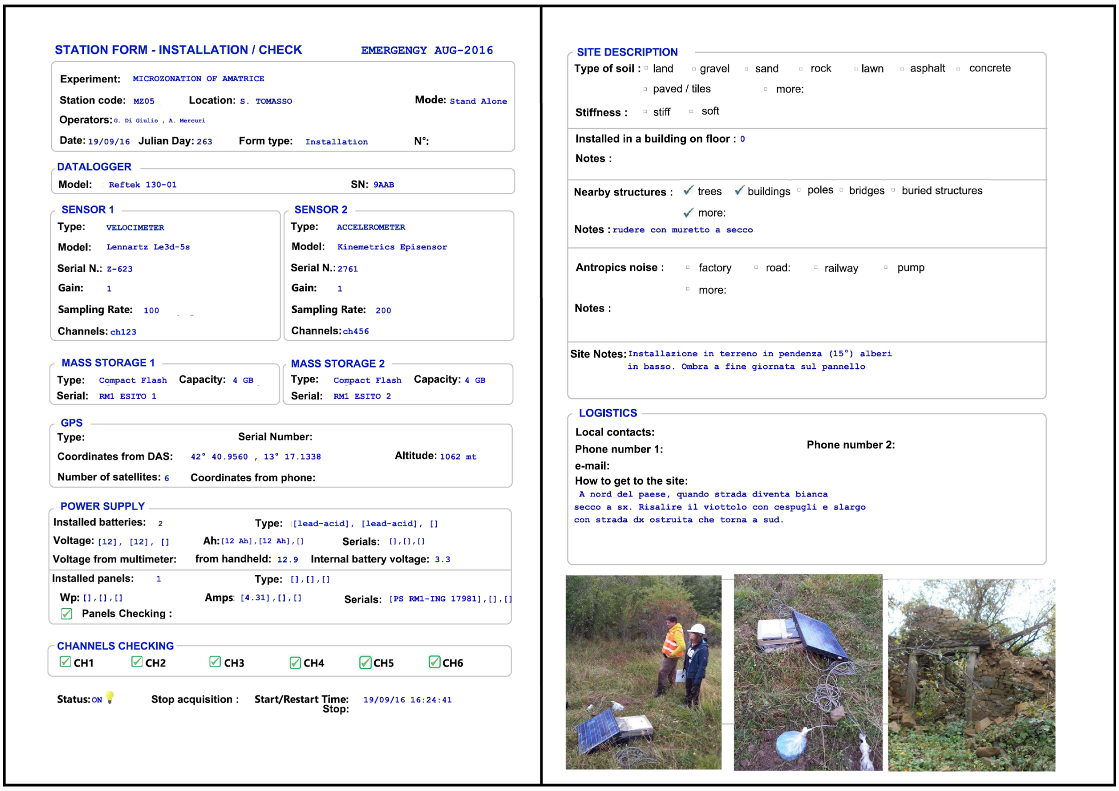

STATION FORM - INSTALLATION / CHECK EMERGENGY AUG-2016

Experiment: MICROZONATION OF AMATRICE
Station code: MZ05 Location: S. TOMASSO Mode: Stand Alone
Operators: G. Di Giulio , A. Mercuri
Date: 19/09/16 Julian Day: 263 Form type: Installation N°:

DATALOGGER
Model: Reftek 130-01 SN: 9AAB

SENSOR 1
Type: VELOCIMETER
Model: Lennartz Le3d-5s
Serial N.: Z-623
Gain: 1
Sampling Rate: 100
Channels: ch123

SENSOR 2
Type: ACCELEROMETER
Model: Kinemetrics Episensor
Serial N.: 2761
Gain: 1
Sampling Rate: 200
Channels: ch456

MASS STORAGE 1
Type: Compact Flash Capacity: 4 GB
Serial: RM1 ESITO 1

MASS STORAGE 2
Type: Compact Flash Capacity: 4 GB
Serial: RM1 ESITO 2

GPS
Type: Serial Number:
Coordinates from DAS: 42° 40.9560 , 13° 17.1338 Altitude: 1062 mt
Number of satellites: 6 Coordinates from phone:

POWER SUPPLY
Installed batteries: 2 Type: [lead-acid], [lead-acid], []
Voltage: [12], [12], [] Ah: [12 Ah], [12 Ah], [] Serials: [], [], []
Voltage from multimeter: from handheld: 12.9 Internal battery voltage: 3.3
Installed panels: 1 Type: [], [], []
Wp: [], [], [] Amps: [4.31], [], [] Serials: [PS RM1-ING 17981], [], []
☑ Panels Checking :

CHANNELS CHECKING
☑ CH1 ☑ CH2 ☑ CH3 ☑ CH4 ☑ CH5 ☑ CH6

Status: ON Stop acquisition : Start/Restart Time: 19/09/16 16:24:41
Stop:

SITE DESCRIPTION
Type of soil : land gravel sand rock lawn asphalt concrete paved / tiles more:
Stiffness : stiff soft

Installed in a building on floor : 0
Notes :

Nearby structures : ✓ trees ✓ buildings poles bridges buried structures ✓ more:
Notes : rudere con muretto a secco

Antropics noise : factory road: railway pump more:
Notes :

Site Notes: Installazione in terreno in pendenza (15°) alberi in basso. Ombra a fine giornata sul pannello

LOGISTICS
Local contacts:
Phone number 1: Phone number 2:
e-mail:
How to get to the site:
A nord del paese, quando strada diventa bianca secco a sx. Risalire il viottolo con cespugli e slargo con strada dx ostruita che torna a sud.

Supplementary Figure 3. Example of field sheet for station MZ05.

The form contains all the relevant information reported during the installation[25], among others: day of year of the measurement, site name, site code, acquisition settings, power setting, serial numbers, check of proper working, etc.

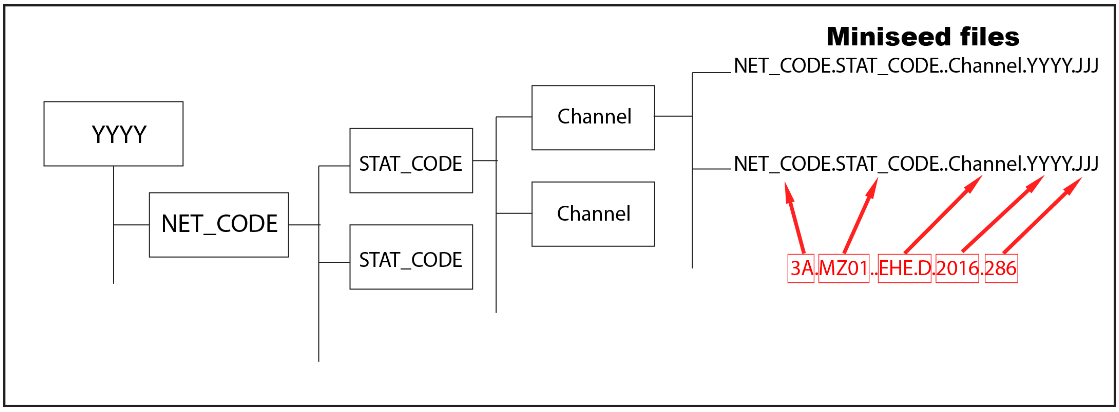

Supplementary Figure 4. Schematic view of the EIDA Archive structure.

The root folder is named as the year of the recordings (2016 in our case), then there is a folder named as the network code (3A) that contains as many folders as the stations are and named as the stations (MZ01, MZ02, and so on). Each of these folders contain three or six folders representative of the recording channels (in our case EHE, EHN, EHZ for the velocimetric data, HNE, HNN, HNZ for the accelerometric data). Finally each channel folder contains the daily miniSEED traces (JJJ means Julian date).

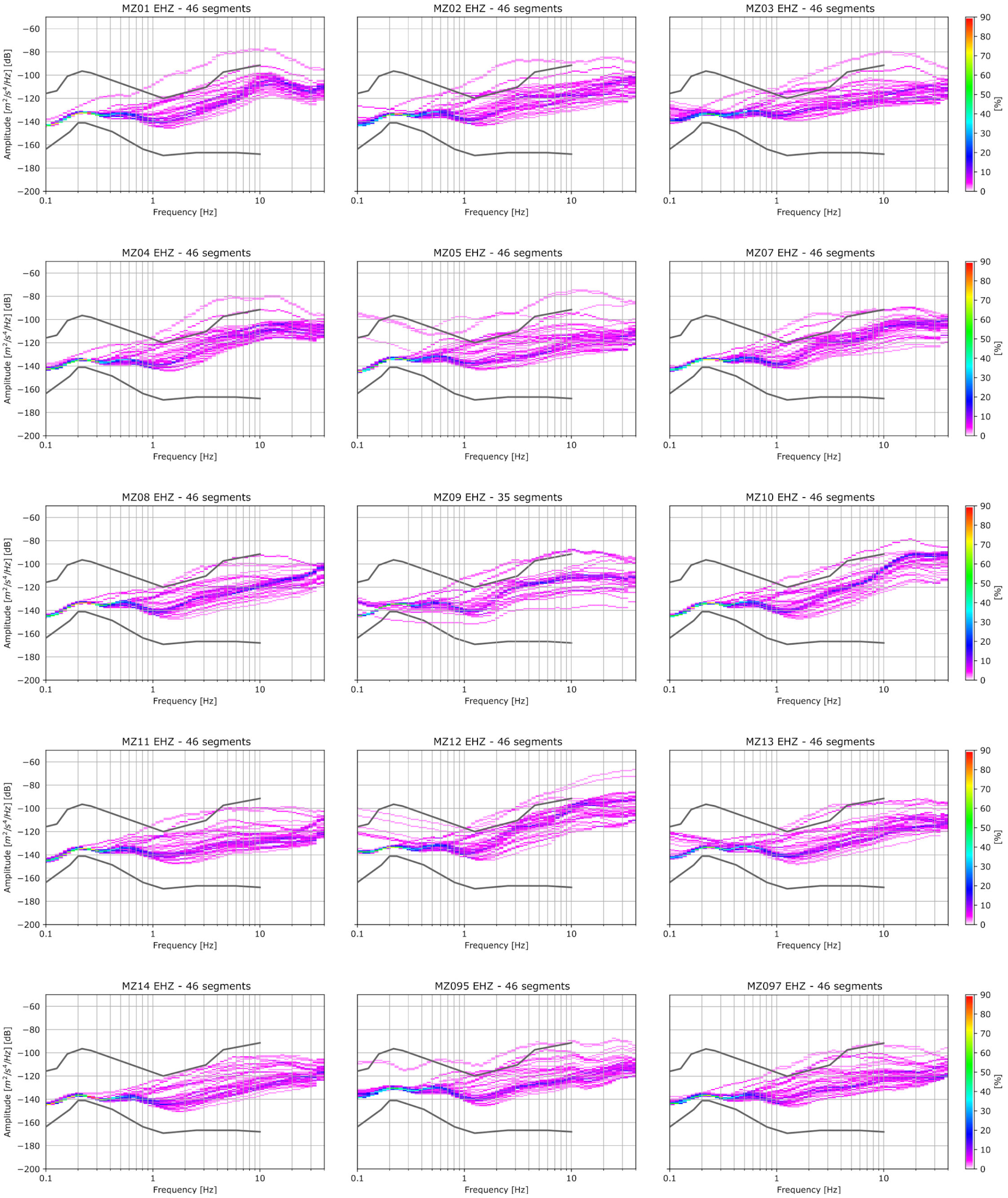

Supplementary Figure 5. PSD on seismic noise.

The black lines are the standard low- (NLNM) and high- (NLHM) noise models[37]. Each colored line is the PSD curve of one of the 24 hours of September 26, 2016. For this analysis we considered only the vertical components of motion (channel EHZ). The color is a representation of the difference between each curve and the average PSD curve. Details on the analysis can be found in [38].
